# PA Mutations Inherited during Viral Evolution Act Cooperatively To Increase Replication of Contemporary H5N1 Influenza Virus with an Expanded Host Range

**DOI:** 10.1128/JVI.01582-20

**Published:** 2020-12-09

**Authors:** Yasuha Arai, Norihito Kawashita, Emad Mohamed Elgendy, Madiha Salah Ibrahim, Tomo Daidoji, Takao Ono, Tatsuya Takagi, Takaaki Nakaya, Kazuhiko Matsumoto, Yohei Watanabe

**Affiliations:** a Department of Infectious Diseases, Graduate School of Medical Sciences, Kyoto Prefectural University of Medicine, Kyoto, Japan; b Faculty of Science and Engineering, Kindai University, Osaka, Japan; c Graduate School of Pharmaceutical Sciences, Osaka University, Osaka, Japan; d Department of Microbiology and Immunology, Faculty of Veterinary Medicine, Damanhour University, Damanhour, Egypt; e The Institute of Scientific and Industrial Research, Osaka University, Osaka, Japan; St. Jude Children’s Research Hospital

**Keywords:** H5N1, evolution, host adaptation, influenza virus

## Abstract

Adaptive mutations and/or reassortments in avian influenza virus polymerase subunits PA, PB1, and PB2 are one of the major factors enabling the virus to overcome the species barrier to infect humans. The majority of human adaptation polymerase mutations have been identified in PB2; fewer adaptation mutations have been characterized in PA and PB1. Clade 2.2.1 avian influenza viruses (H5N1) are unique to Egypt and generally carry the human adaptation PB2-E627K substitution during their dissemination in nature. In this study, we identified other human adaptation polymerase mutations by analyzing phylogeny-associated PA mutations that H5N1 clade 2.2.1 viruses have accumulated during their evolution in the field. This analysis identified several PA mutations that produced increased replication by contemporary clade 2.2.1.2 viruses *in vitro* in human cells and *in vivo* in mice compared to ancestral clade 2.2.1 viruses. The PA mutations acted cooperatively to increase viral polymerase activity and replication in both avian and human cells, with the effect being more prominent in human cells at 33°C than at 37°C. These results indicated that PA mutations have a role in establishing contemporary clade 2.2.1.2 virus infections in poultry and in adaptation to infect mammals. Our study provided data on the mechanism for PA mutations to accumulate during avian influenza virus evolution and extend the viral host range.

**IMPORTANCE** Clade 2.2.1 avian influenza viruses (H5N1) are unique to Egypt and have caused the highest number of human H5N1 influenza cases worldwide, presenting a serious global public health threat. These viruses may have the greatest evolutionary potential for adaptation from avian hosts to human hosts. Using a comprehensive phylogenetic approach, we identified several novel clade 2.2.1 virus polymerase mutations that increased viral replication *in vitro* in human cells and *in vivo* in mice. These mutations were in the polymerase PA subunit and acted cooperatively with the E627K mutation in the PB2 polymerase subunit to provide higher replication in contemporary clade 2.2.1.2 viruses than in ancestral clade 2.2.1 viruses. These data indicated that ongoing clade 2.2.1 dissemination in the field has driven PA mutations to modify viral replication to enable host range expansion, with a higher public health risk for humans.

## Abstract (precis)

Clade 2.2.1 avian influenza viruses (H5N1) are unique to Egypt and have caused the highest number of human H5N1 influenza cases worldwide, presenting a serious global public health threat. These viruses may have the greatest evolutionary potential for adaptation from avian hosts to human hosts. Using a comprehensive phylogenetic approach, we identified several novel clade 2.2.1 virus polymerase mutations that increased viral replication *in vitro* in human cells and *in vivo* in mice. These mutations were in the polymerase PA subunit and acted cooperatively with the E627K mutation in the PB2 polymerase subunit to provide higher replication in contemporary clade 2.2.1.2 viruses than in ancestral clade 2.2.1 viruses. These data indicated that ongoing clade 2.2.1 dissemination in the field has driven PA mutations to modify viral replication to enable host range expansion, with a higher public health risk for humans.

## INTRODUCTION

The H5N1 subtype of highly pathogenic avian influenza (AI) viruses was first reported in Egypt in 2006 and has been enzootic since then (1, 2). H5N1 viruses in Egypt have formed their own clade, clade 2.2.1, with 4th-order clades 2.2.1.1 and 2.2.1.2. Clade 2.2.1.2 strains have dominated in the field and have been prevalent in a wide range of poultry. Egypt has had the highest number of cases of human H5N1 infections worldwide (41.7%), as of 8 May 2020 according to the WHO, and is now regarded as a hot spot for H5N1 evolution with increased bird-to-human transmission (1, 2). In addition, H9N2 and H5N8 AI viruses have been cocirculating in Egypt and have become endemic in poultry since 2011 and 2017, respectively, with four human H9N2 infection cases reported (3, 4). The continuous dissemination of these AI viruses raises concern for public health and animal health in the Middle East (and also worldwide), with the potential risk of the emergence of novel AI viruses (5, 6).

Influenza virus replication is mediated by a trimeric polymerase complex composed of the viral PB2, PB1, and PA proteins. The polymerase complex binds viral RNA (vRNA) and NP to form a ribonucleoprotein. For human infections, the polymerase complex needs to adapt to replicate in human cells through gene mutation or reassortment (7, 8). In the past, the majority of polymerase gene mutations have been found in the PB2 gene. The PB2-E627K substitution is the most common influenza virus human adaptation mutation and is present in most seasonal human influenza viruses except the 2009 pandemic H1N1 virus. The PB2-E627K mutation has been found in several H5N1 and H7N9 viruses that have infected humans (9–11). It also has been correlated with the increased virulence of H5N1 virus in mice and enabled the virus to replicate efficiently in the human upper respiratory tract (9, 12), where the temperature is lower than the core body temperature. Other mammal adaptation mutations include PB2-D701N, -K526R, -Q591K, -E192K, -K702R, and -E627V (10, 13–17).

In contrast to PB2, there has been limited information about human adaptation mutations in PB1 and PA (18, 19). Recent studies reported that the emergence of the 2009 pandemic H1N1 (H1N1 pdm09) virus, which had avian-origin PB2 and PA genes, was linked to the acquisition of PA mutations (20–22), indicating a key role for PA in AI virus adaptation during pandemic virus emergence. However, the precise function(s) of PA adaptive mutations has been less defined, which has prevented an understanding of the molecular mechanism(s) for influenza virus human adaptation.

AI viruses circulating in Egypt have a characteristic feature: they have accumulated representative human adaptation mutations during their dissemination in bird populations in the field. The Egyptian H5N1 clade 2.2.1 strains in poultry species generally carry the PB2-E627K mutation (18, 23, 24). In addition, we recently reported that the H9N2 virus in the Middle East (G1-A/B subclade), centered in Egypt, commonly carries the PB2-E627V, -E543D, -A655V, and -K526R mutations, which all contributed to more fit replication in human cells and mice (13). These results contrasted those for viruses isolated in Asia, where representative human adaptation mutations (e.g., PB2-E627K and -K526R) were found frequently in viruses from AI virus-infected patients (10, 25) but not in field AI strains, with a few exceptions. Thus, it is of great importance to monitor the evolution of Middle Eastern AI viruses that can accumulate mutations to potentially expand their host range.

In this study, we investigated the phylogeny of mutations in PA, the polymerase subunit that increased the polymerase activity of contemporary H5N1 clade 2.2.1.2 viruses, and the effect of these mutations on the biological properties of the H5N1 virus, including its adaption to infect mammals. Our results provide data for understanding how PA mutations accumulate during AI virus evolution to affect the viral host range.

## RESULTS

### Polymerase activity of ancestral and contemporary H5N1 viruses isolated in Egypt.

**FIG 1:**
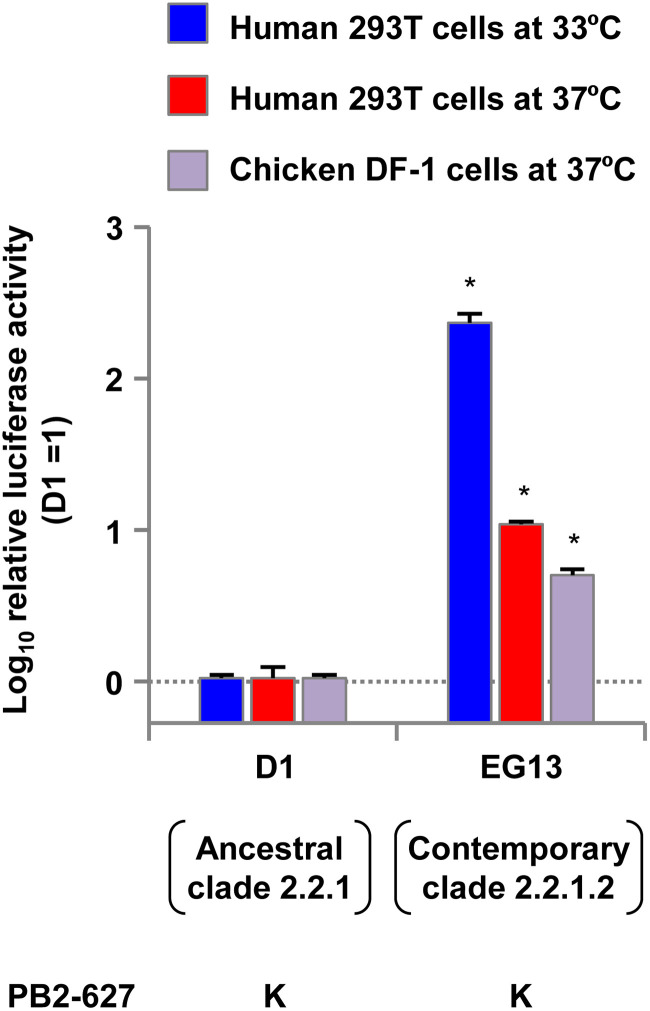
Polymerase activity of ancestral and contemporary clade 2.2.1 viruses. Viral polymerase activity was measured by minigenome assays in human 293T cells at 33°C and 37°C and in avian DF-1 cells at 37°C. The data are expressed relative to the results for the ancestral D1 virus. Each data point is the mean ± standard deviation (SD) from three independent experiments. Asterisks indicate a *P* value of <0.01.

In our previous study, we isolated H5N1 clade 2.2.1.2 viruses from chickens in Egypt in 2013 and found that one of the viruses, A/chicken/Egypt/CL69/2013 (denoted EG13 here), replicated at a high level in human cells and with high pathogenicity in mice (6). To investigate the polymerase activity of ancestral (i.e., 2006–2009 isolates) and contemporary (i.e., 2010–2015 isolates) H5N1 viruses, we compared the polymerase activity of EG13 with that of A/duck/Egypt/D1Br/2007 (denoted D1 here). The EG13 and D1 viruses are representative contemporary and ancestral clade 2.2.1 viruses, respectively, and their properties have been characterized in previous studies (6, 24), which motivated us to use these two viruses in this study. As with most clade 2.2.1 viruses, both the EG13 and D1 viruses carry the PB2-E627K mutation. In this study, minigenome assays were carried out at both 33°C and 37°C in human 293T cells, the temperatures of the human upper and lower respiratory tracts, respectively, and at 37°C in avian DF-1 cells to allow the results of these studies to be compared with those for 293T cells at 37°C, as reported previously (18, 19). The EG13 contemporary virus had significantly higher polymerase activity than the D1 ancestral virus in both 293T cells and DF-1 cells (Fig. 1): the difference was greater in human cells at 33°C, where EG13 had >2-log-higher polymerase activity. Clade 2.2.1 and descendant clade 2.2.1.2 viruses have had no reassortment event during their evolution in Egypt (26). This suggested that a mutation(s) in polymerase-associated genes, other than PB2-E627K, accumulated in EG13 to produce its higher polymerase activity.

### Effect of the PA gene on EG13 polymerase activity in human and avian cells.

**FIG 2:**
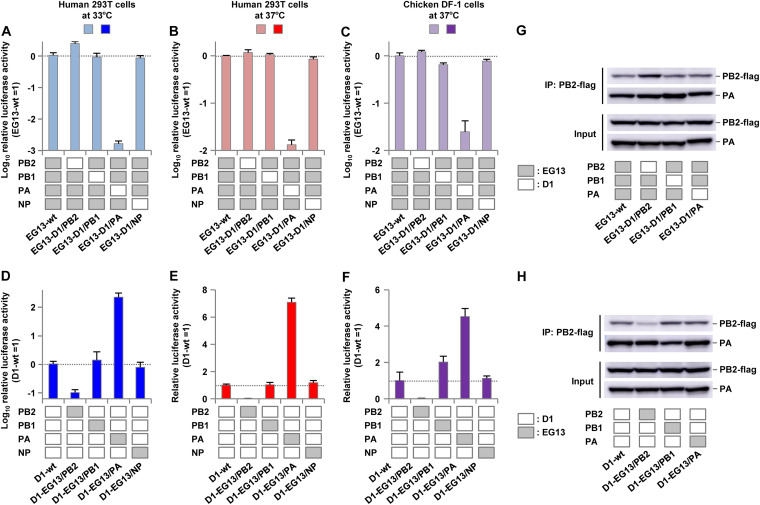
Polymerase activity of reassortant polymerases formed by subunits from ancestral and contemporary clade 2.2.1 viruses. The viral polymerase activity of reassortant polymerases formed by subunits from ancestral D1 and contemporary EG13 viruses was measured by minigenome assays in human 293T cells at 33°C and 37°C and in avian DF-1 cells at 37°C. (A to C) Polymerase activity of reassortant polymerase complexes composed of one subunit from the D1 virus and the other subunits from the EG13 virus. The data are expressed relative to the results for wild-type EG13. (D to F) Polymerase activity of reassortant polymerase complexes composed of one subunit from the EG13 virus and the other subunits from the D1 virus. The data are expressed relative to the results for wild-type D1. Each data point is the mean ± SD from three independent experiments. (G and H) Western blot analysis of reassortant polymerase complexes without (Input) and with (IP: PB2-flag) immunoprecipitation with PB2-Flag. (G) Reassortant polymerase complexes composed of one subunit from the D1 virus and the other subunits from the EG13 virus. (H) Reassortant polymerase complexes composed of one subunit from the EG13 virus and the other subunits from the D1 virus. Representative images are shown.

To determine the effect of each polymerase subunit on EG13 polymerase activity, the activity of polymerase complexes, in which the polymerase subunits were reassorted between the EG13 and D1 viruses, was measured by minigenome assays in 293T and DF-1 cells. In the EG13 background, PA from the D1 virus (denoted D1/PA here) significantly decreased the viral polymerase activity in both human cells and avian cells, but the D1/PB2 reassortant had only a minimal increase in polymerase activity (Fig. 2A to C). In contrast, in the D1 background, EG13/PA significantly increased the polymerase activity in both human cells and avian cells, but EG13/PB2 reduced the polymerase activity in these cells (Fig. 2D to F). These effects were greater in human cells at 33°C than at 37°C and also greater in human cells than in avian cells.

To investigate the effect of each subunit on complex formation of the reassorted polymerases in this study, the reassortant polymerase complexes were analyzed by Western blotting with and without immunoprecipitation of PB2-Flag. Similar amounts of PA in the polymerase complexes were coprecipitated with PB2 (Fig. 2G and H), confirming that reassortment did not noticeably affect the formation of the trimeric polymerase complex. These results suggested that the high polymerase activity of EG13 was mainly due to its PA gene, which appeared to have a direct effect on polymerase function but not on the formation of the polymerase complex.

### Accumulation of mutations in the clade 2.2.1 virus PA gene during viral evolution in Egypt.

**FIG 3:**
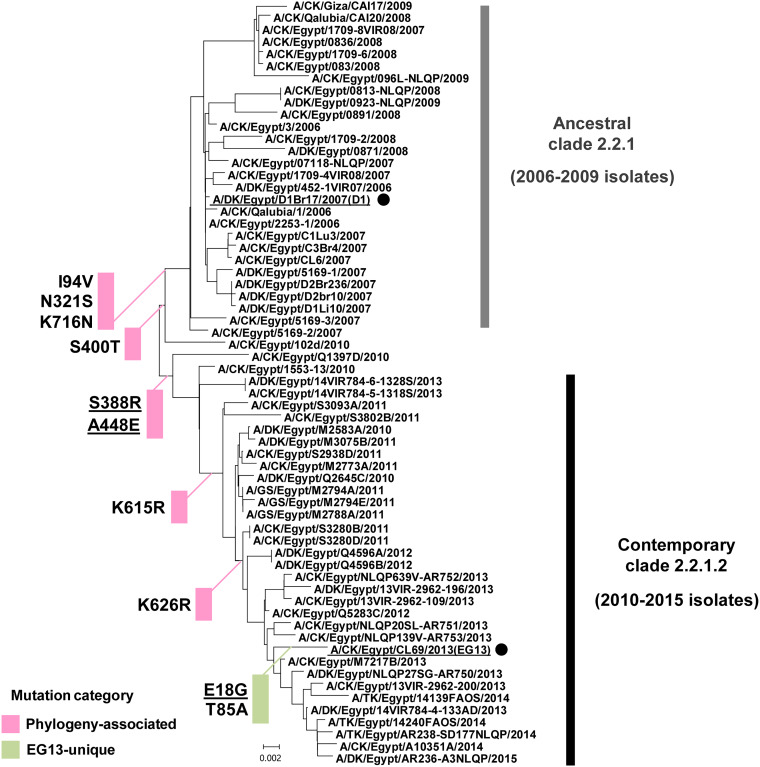
Phylogeny of the PA gene in clade 2.2.1 viruses isolated in Egypt. The phylogenetic tree of the PA genes of ancestral clade 2.2.1 viruses isolated in Egypt, including the D1 virus, and of contemporary clade 2.2.1.2 viruses isolated in Egypt, including the EG13 virus, was reconstructed from the nucleotide sequences of the PA genes of the Egyptian reference strains in the GISAID database. This reconstruction used the neighbor-joining method with 1,000 bootstrap replicates and was rooted to the prototype A/quail/Hong Kong/G1/1997 (H5N1) strain. The two clade 2.2.1 strains in this study are representative ancestral (D1) and contemporary (EG13) strains and are underlined and marked with black circles. CK, DK, GS, and TK in the virus strain names denote chicken, duck, goose, and turkey hosts, respectively. The PA mutations acquired during the evolution of clade 2.2.1 viruses are shown beside each branch, with the mutations grouped into two categories, i.e., 8 phylogeny-associated mutations and 2 EG13-unique mutations. The three mutations that were shown to act cooperatively in this study to increase clade 2.2.1.2 replication are underlined.

To trace the evolution of the PA gene in clade 2.2.1 viruses, we analyzed the phylogeny of PA genes in ancestral clade 2.2.1 viruses (i.e., 2006–2009 isolates, including the D1 virus) and contemporary viruses (i.e., 2010–2015 isolates that were mostly of clade 2.2.1.2, including the EG13 virus) (Fig. 3). The viral PA sequences were analyzed, relative to the ancestral D1 sequence, to search for mutations that contemporary clade 2.2.1.2 viruses had accumulated during their evolution in the field. A total of 10 amino acid substitutions were identified. These formed two mutation groups: one was a group of eight mutations that were in almost all the contemporary viruses isolated after 2010, and the other was a group of two mutations that were unique to the EG13 virus. These results suggested that the phylogeny-associated PA mutations may have been involved in the establishment of contemporary clade 2.2.1.2 viruses in poultry, with strain-specific mutations possibly affecting the traits of these strains.

### Effect of PA mutations on the polymerase activity of clade 2.2.1 viruses in human cells.

**FIG 4:**
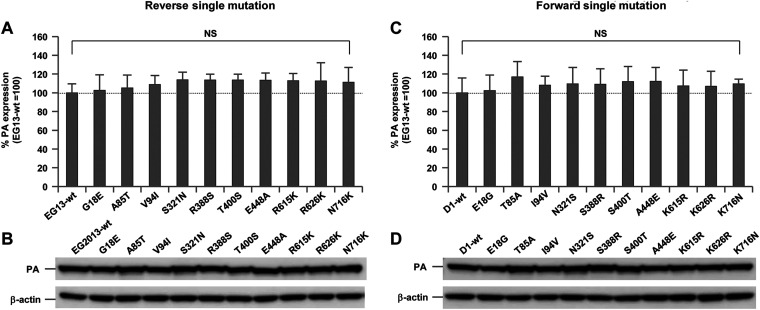
Quantification of PA expression. 293T cells were transfected with PA expression plasmids carrying the indicated mutations, and cell lysates were analyzed by Western blotting using anti-PA antibody. (A and C) After quantification of the band intensity, the amount of PA produced by each mutant was expressed relative to that for wild-type PA. (A) Expression of contemporary EG13-PA, each carrying a reverse PA mutation. (C) Expression of ancestral D1-PA, each carrying a forward PA mutation. Each data point is the mean ± SD from five independent experiments. NS indicates no statistically significant difference. (B and D) Representative images of the Western blots of each EG13-PA with a reverse mutation (B) and each D1-PA with a forward mutation (D).

Each of the substitution mutations that were identified in the PA gene was introduced into the EG13/PA gene as a reverse mutation and into the D1/PA gene as a forward mutation. All these mutants had similar levels of PA expression (Fig. 4A to D), indicating that these mutations had little effect on PA expression.

**FIG 5:**
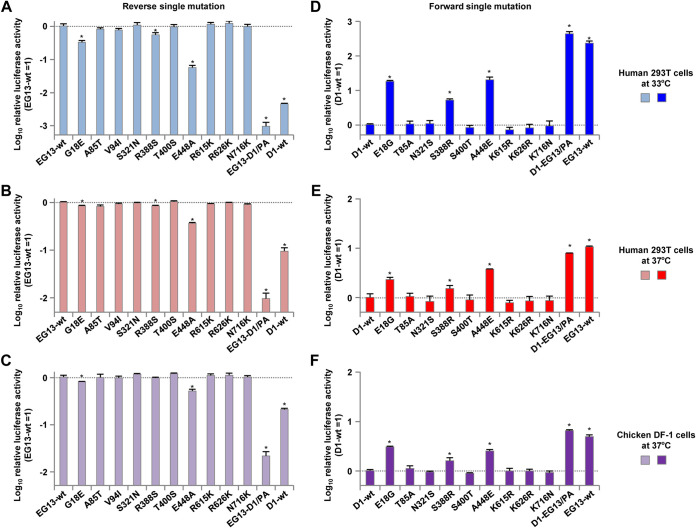
Effect of PA mutations on the polymerase activity of clade 2.2.1 viruses in human and avian cells. (A to C) Polymerase activity of contemporary EG13 viruses, each carrying PA with the indicated reverse mutation, measured by minigenome assays in human 293T cells at 33°C (A) and 37°C (B) and in avian DF-1 cells at 37°C (C). The data are expressed relative to the results for wild-type EG13. (D to F) Polymerase activity of ancestral D1 viruses, each carrying PA with the indicated forward mutation, measured by minigenome assays in human 293T cells at 33°C (D) and 37°C (E) and in avian DF-1 cells at 37°C (F). The data are expressed relative to the results for wild-type D1. Each data point is the mean ± SD from five independent experiments. Asterisks indicate a *P* value of <0.01.

To investigate the effect of the PA mutations on polymerase activity, we measured the activity of the polymerase complexes, each with a PA subunit carrying one of the mutations, using minigenome assays in DF-1 cells and 293T cells. In the EG13 background, the G18E, R388S, and E448A reverse mutations each decreased the polymerase activity, although the decrease was greatest with the reassorted D1/PA (denoted EG13-D1/PA here) that was included as a control (Fig. 5A to C). The V94I substitution was excluded from subsequent studies because it had little effect on polymerase activity and because of the similarity of the Val and Ile side chains, which differed from other substitutions (Fig. 5A to C). In contrast, in the D1 background, the E18G, S388R, and A448E forward mutations each increased the polymerase activity by up to >1 log in human cells at 33°C, but the increase was greater with the reassorted EG13 PA subunit that was included as a control (denoted D1-EG13/PA here) (Fig. 5D to F). The effects of the single mutations that affected polymerase activity were greatest in human cells at 33°C, less in human cells at 37°C, and smallest in avian cells at 37°C.

### The PA-E18G, -S388R, and -A448E mutations act cooperatively to increase the polymerase activity of clade 2.2.1.2 viruses.

Based on the effects of the single mutations, we selected three PA substitutions (i.e., E18G, S388R, and A448E) for further study to investigate whether they might act cooperatively on polymerase activity. Multiple mutations were combined in their putative phylogenetic order, i.e., the G18E, R388S, and E448A reverse mutations and the A448E, S388R, and E18G forward mutations. The R388S/E448A reverse mutations were also included in this study because they were associated with the high polymerase activity in ancestral clade 2.2.1 viruses described below.

**FIG 6 F6:**
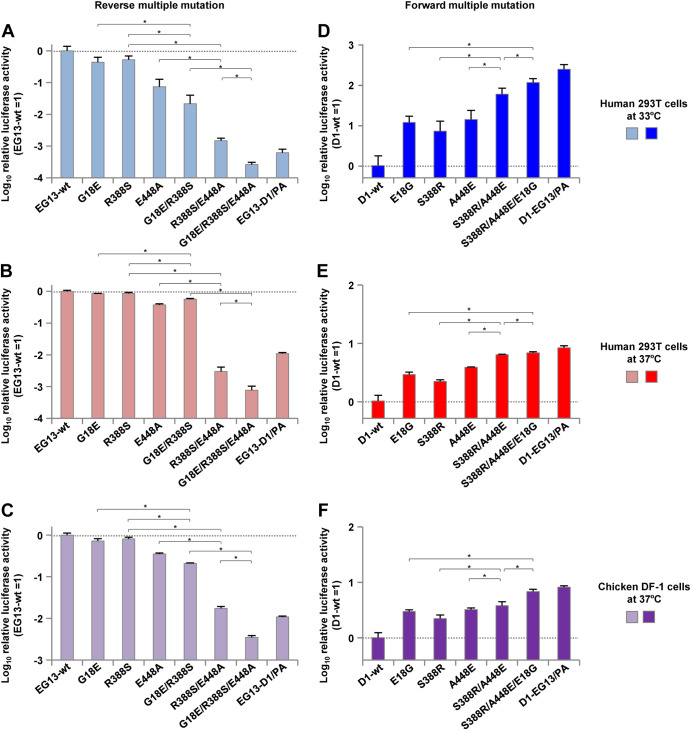
Cooperative effects of PA mutations on clade 2.2.1 polymerase activity in human and avian cells. (A to C) Polymerase activity of contemporary EG13 viruses, each carrying the indicated PA reverse mutation, singly or in combination, measured by minigenome assays in human 293T cells at 33°C (A) and 37°C (B) and in avian DF-1 cells at 37°C (C). The data are expressed relative to the results for wild-type EG13. (D to F) Polymerase activity of ancestral D1 viruses, each carrying the indicated PA forward mutation, singly or in combination, measured by minigenome assays in human 293T cells at 33°C (D) and 37°C (E) and in avian DF-1 cells at 37°C (F). The data are expressed relative to the results for wild-type D1. Each data point is the mean ± SD from five independent experiments. Asterisks indicate a *P* value of <0.01.

The three reverse mutations acted cooperatively to decrease EG13 polymerase activity by >3 logs in human cells (Fig. 6A to C), with a greater effect at 33°C than at 37°C. The polymerase with the G18E/R388S/E448A triple mutation had the lowest activity, with a level similar to that of EG13-D1/PA: the R388S/E448A double mutation produced the greatest reduction in polymerase activity. The relative polymerase activities were wild-type EG13 (EG13-wt) > G18E > G18E/R388S > R388S/E448A > G18E/R388S/E448A. In contrast, the forward mutations acted cooperatively to increase D1 polymerase activity in human cells by >2 logs at 33°C and nearly 1 log at 37°C (Fig. 6D to F). The S388R/E448A/G18E triple mutation had the highest polymerase activity, with a level comparable to that of D1-EG13/PA: the greatest increase was produced by the S388R/A448E mutation. The relative polymerase activities were D1-wt < A448E < A448E/S388R < A448E/S388R/E18G. In avian cells, the three mutations had an appreciably attenuated effect in the EG13 background, although the distinct effect between the polymerase activity in human cells and that in avian cells was not noticeable in the D1 background. These results suggested that the phylogeny-associated A448E and S388R mutations acted cooperatively to increase the polymerase activity of the contemporary clade 2.2.1.2 viruses that have been circulating since 2010, with the effect being most prominent in infected human cells at 33°C. In addition, the E18G strain-specific mutations had a collateral effect on the increase of EG13 polymerase activity.

### Effect of the PA-A448E, -S388R, and -E18G mutations on the replication of clade 2.2.1.2 viruses in human cells.

**FIG 7:**
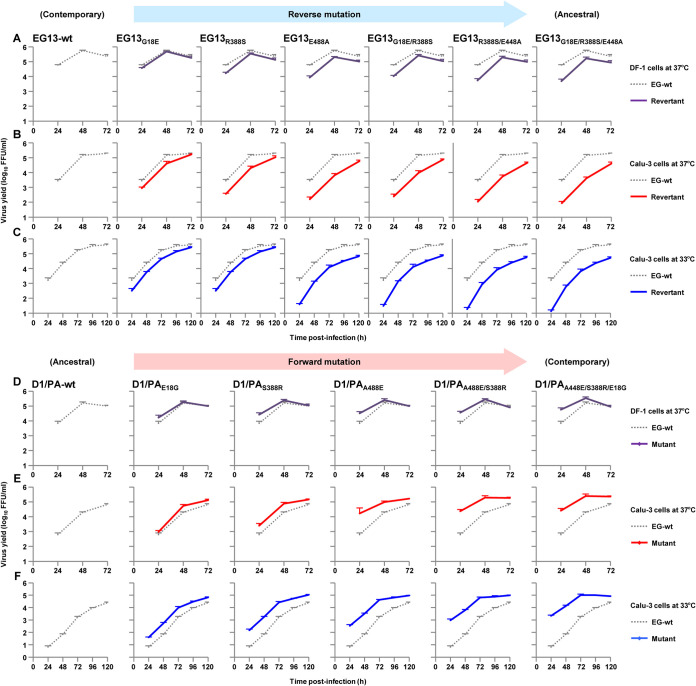
Replication kinetics of EG13 and D1 viruses carrying PA mutations in human and avian cells. (A to C) Avian DF-1 cells (A) and human Calu-3 cells (B and C) were infected with the EG13-wt virus or an EG-13 virus carrying the indicated PA mutant at MOIs of 0.003 and 0.03, respectively, and incubated at 37°C (A and B) and 33°C (C). (D to F) Avian DF-1 cells (D) and human Calu-3 cells (E and F) were infected with the reassorted D1/PA viruses carrying PA-wt or PA with the indicated mutation, PB2, PB1, and NP from EG13, and the other genes from D1, at MOIs of 0.003 and 0.03, respectively, and incubated at 37°C (D and E) or 33°C (F). At the indicated times postinfection, the progeny virus titers were measured by FFU assays. Each data point is the mean ± SD from three independent experiments.

We investigated the effect of the PA-A448E, -S388R, and -E18G substitution mutations on the replication kinetics of clade 2.2.1 viruses using recombinant viruses carrying these mutations. Avian DF-1 cells and human airway epithelial Calu-3 cells were infected with EG13 viruses carrying the PA reverse mutations, and the viral replication kinetics was measured. In both cell types, the PA mutants produced lower progeny virus yields than wild-type EG13 virus (Fig. 7A to C), with the greatest effects in Calu-3 cells at 33°C. PA with the triple reverse mutation G18E/R388S/E448A (denoted EG13_G18E/R388S/E448A_ here) had the lowest progeny virus yield in both cell lines, in particular by >2 logs in human cells at early times postinfection at 33°C, with the R388S/E448A mutation producing the greatest effect.

The effect of the forward PA mutations in the D1 background on viral replication was also studied. For these studies, we used a recombinant D1 virus carrying a reassorted polymerase complex composed of D1/PA with the other genes (PB2, PB1, and NP) from EG13 (denoted D1/PA virus here) to assess PA-specific effects on viral replication. This experimental strategy was chosen since previous studies suggested that influenza virus polymerase genes may coevolve in the field (18, 27, 28). Therefore, we used EG13-PB2 and -PB1 together with D1-PA carrying the three mutations since the mutant D1-PA should be similar to EG13-PA in terms of function. This choice of polymerase subunits should have minimized any potential incompatibility of the D1 and EG13 polymerase genes, although no noticeable mismatch between the two viruses was observed in polymerase complex formation and minigenome assays. Compared to wild-type D1/PA virus, D1/PA viruses carrying the forward PA mutations produced higher progeny virus yields in Calu-3 cells throughout the postinfection time in this study (Fig. 7D to F), with more significant effects at 33°C. However, in DF-1 cells, the PA mutants had a limited effect, with higher progeny virus yields only at the early time points postinfection. The triple mutant D1/PA_A448E/S388E/E18G_ had the greatest progeny virus yield in both cells: D1/PA-wt < A448E < A448E/S388R < A448E/S388R/E18G, which was comparable to that of wild-type EG13.

Together, these results suggested that during the evolution of contemporary clade 2.2.1.2 viruses, the A448E and S388R mutations acted cooperatively to provide a more fit phenotype for replication in human cells at a lower temperature and that the E18G mutation produced strain-specific higher replication in EG13.

### Effect of the PA-A448E, -S388R, and -E18G mutations on the replication and virulence of clade 2.2.1 viruses *in vivo* in mice.

To assess the relevance of the *in vitro* effects of the A448E/S388R/E18G mutation to *in vivo* infections, BALB/c mice were inoculated intranasally with serial dilutions of D1/PA-wt virus and D1/PA viruses carrying representative forward mutants (i.e., D1/PA_A448E_, D1/PA_A448E/S388R_, and D1/PA_A448E/S388R/E18G_) or with EG13-wt virus and the EG13_G18E/R388S/E448A_ reverse mutant. The body weight and survival of each infected mouse were monitored for 2 weeks.

**FIG 8:**
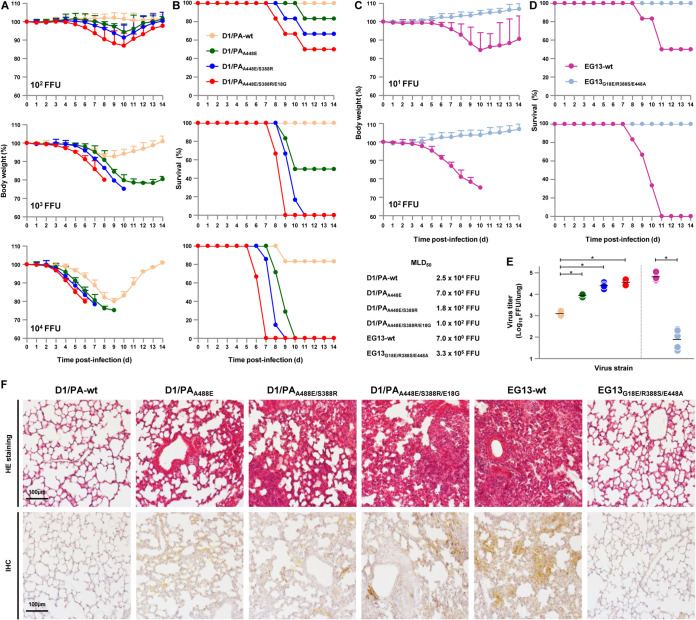
Virulence and replication of D1 and EG13 viruses carrying PA mutations in the respiratory tract of infected mice. (A to D) Four- to five-week-old BALB/c mice (6 mice per group) were inoculated intranasally with 10^1^ to 10^5^ FFU of the indicated PA mutants in the D1/PA genetic background (A and B) or the EG13 genetic background (C and D). (A and C) The body weight of the infected mice was monitored daily for 14 dpi. The mean ± SD of the percentage of the initial body weight for each group of mice is shown. (B and D) Survival of the infected mice. Survival was calculated, including mice that were humanely sacrificed after they had lost more than 30% of their body weight within a few days. (E) Viral titers in the lungs of mice (6 mice per group) infected with 10^3^ FFU of the EG13 and G1/P viruses carrying the indicated PB2 mutations at 6 dpi. Each symbol marks the titer in an individual mouse. Asterisks indicate a *P* value of <0.01. (F) Representative photomicrographs of hematoxylin and eosin (HE)-stained (top) and immunohistochemically (IHC) stained (bottom) lung sections from mice infected with the indicated viruses at 6 dpi. In the immunohistochemically stained tissues, the viral antigen was stained deep brown on a hematoxylin-stained background.

A dose of 10^3^ focus-forming units (FFU) of wild-type D1/PA virus caused slight weight loss in infected mice, and all 6 of the infected mice survived (Fig. 8A and B), indicating a limited ability of the virus carrying PB2-E627K to replicate in mice: the 50% mouse lethal dose (MLD_50_) was 2.5 × 10^4^ FFU, which was in agreement with the MLD_50_ of ancestral clade 2.2.1 viruses reported previously (24, 29). In contrast, the same dose of PA carrying the forward mutants caused dramatic weight loss: 3 of 6 mice infected with D1/PA_A448E_ survived, and none of the 6 mice infected with D1/PA_A448E/S388R_ or D1/PA_A448E/S388R/E18G_ survived, with death being faster for D1/PA_A448E/S388R/E18G_-infected mice than for D1/PA_A448E/S388R_-infected mice. D1/PA_A448E/S388R_ and D1/PA_A448E/S388R/E18G_ had MLD_50_ values of 1.8 × 10^2^ FFU and 1.0 × 10^2^ FFU, respectively, which were >2 logs lower than that of wild-type D1/PA.

The EG13-wt contemporary virus had a highly virulent phenotype, with an MLD_50_ of 7.0 × 10^0^ FFU, whereas the EG13_G18E/R388S/E448A_ reverse mutant had a markedly attenuated phenotype, with an MLD_50_ of 3.3 × 10^5^ FFU (Fig. 8C and D), which corresponded to the MLD_50_ of wild-type D1 reported previously (24).

To determine the virus titers in infected mice, groups of 6 mice were inoculated intranasally with 10^3^ FFU of each virus. Lungs were collected at 6 days postinfection (dpi), and virus titers in the lungs were measured by focus-forming assays. Virus titers in the infected mice were consistent with their polymerase activity and replication kinetics in human cells *in vitro* (Fig. 5 [Fig F6]to 7). PA forward mutants replicated more efficiently in the mouse lungs, with titers of D1/PA_A448E/S388R(/E18G)_ being comparable to those of contemporary EG13 (Fig. 8E). In contrast, the EG13_G18E/R388S/E448A_ reverse mutant replicated with an appreciably lower titer in the mouse lungs.

The lungs of infected mice were examined by histopathology at 6 dpi. Ancestral D1/PA-wt and the EG13_G18E/R388S/E448A_ reverse mutant caused only a limited inflammatory response in the lungs of infected mice (Fig. 8F, top). In contrast, the PA forward mutants and contemporary EG13-wt induced more severe bronchiolar necrosis and alveolitis, characterized by hemorrhage and inflammatory cell infiltrates. The most severe pathological changes were detected in the lungs of mice infected with D1/PA_A448E/S388R(/E18G)_ or EG13-wt. The degree of pathological change corresponded to the amount of H5 antigen detected by immunohistochemistry in the alveolar areas of the lungs (Fig. 8F, bottom).

### Effect of PA mutations on structural changes in the polymerase complex of clade 2.2.1 viruses.

**FIG 9:**
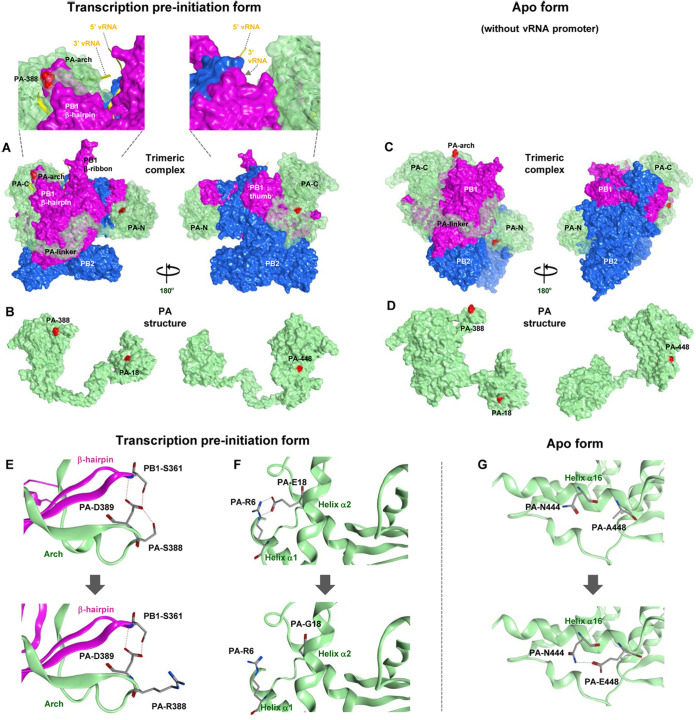
Structural model of the clade 2.2.1 polymerase complex. Shown is a structural model of the D1/PA heterotrimeric polymerase complex. (A and C) Transparent surface diagrams of the D1/PA polymerase complex composed of PB2 (blue), PB1 (pink), and PA (green) with the A448E, S388R, and E18G mutations (red). (A) The transcription preinitiation form (PDB accession number 6RR7 as the modeling template), where the polymerase complex binds to the vRNA promoter (yellow). A close-up of the polymerase complex around the vRNA promoter is also shown. (C) The apo form (without the vRNA promoter) (PDB accession number 6QPF as the modeling template). (B and D) Surface views of the PA structure in the transcription preinitiation form (B) and the apo form (D). The left and right PA structures differ by 180° in orientation. (E to G) Polymerase structure without (top) and with (bottom) the indicated PA mutations in the D1/PA heterotrimeric polymerase complex with vRNA. (E) Close-up of residue 388 in the D1/PA polymerase complex. (F) Close-up of residue 18 in the D1/PA polymerase complex. (G) Close-up of residue 448 in the D1/PA polymerase complex. Potential interactions between residues are represented by broken lines.

To investigate the structural basis for the increased clade 2.2.1 polymerase activity and replication by the PA mutations identified in this study (i.e., A448E, S388R, and E18G), we generated models of the D1/PA virus trimeric polymerase complex structure. The influenza virus polymerase complex is inherently flexible and can adopt several conformations depending on the presence or absence of specific viral RNA (30–32). Our models showed that the three PA mutations were exposed on the D1/PA surface in both polymerase complex conformations that we examined (Fig. 9A to D). In the transcription preinitiation form (PDB accession number 6RR7 as the modeling template), A448E, S388R, and E18G were located on the outer side of the polymerase complex (Fig. 9A and B). PA-S388 was located on a PA arch domain close to a PB1 β-hairpin domain, both of which have been reported to form a vRNA contact site (33). There were hydrogen bond bridges between PA-S388, PA-D389, and PB1-S361 in the PB1 β-hairpin, but the S388R substitution obviated this bridge, presumably affecting interactions with the RNA 5′ promoter (Fig. 9E). PA-E18 was located on helix α2 and contacted PA-R6 on the apposed helix α1, but the E18G substitution obviated this interaction, probably reducing structural stability at this position (Fig. 9F). In the apo form of the polymerase complex (PDB accession number 6QPF as the modeling template), our model indicated that PA-A448E, located on helix α16, created a hydrogen bond to PA-N444 (Fig. 9G), presumably stabilizing this helix structure and affecting its interaction with PB1. These results suggested that the multiple mutations emerged coordinately to optimize the polymerase complex structure for improved replication in human hosts.

## DISCUSSION

Adaptive mutations and/or reassortments in the avian influenza virus polymerase complex are major factors enabling the virus to overcome the species barrier between birds and humans (2, 7, 34). The avian PB1 polymerase subunit can function in human cells when combined with human-adapted PB2 and PA polymerase subunits (35). This was observed in the 1957 and 1968 pandemic viruses that contained an avian PB1 gene combined with PB2 and PA genes from human-adapted viruses (36). PB2 was the first polymerase subunit that was found to carry human adaptation mutations affecting polymerase activity. PB2-E627K is the most characterized human adaptation mutation. A recent study showed that low polymerase activity due to PA in the trimeric polymerase complex can drive the PB2-E627K substitution in human cells (37), implying a functional association of PB2 and PA during AI virus adaptation in mammals.

Recently, it has become apparent that PA plays a role in the host adaptation of AI viruses (35, 38), with mutations identified throughout the PA gene sequence (i.e., A36T, S37A, T85I, G186S, L336M, A343S/T, K356R, N383D, and N409S) (19, 20, 22, 39–43). These mutations imply that PA plays multiple roles in AI virus adaptation. However, the underlying adaptation mechanism(s) remains largely unclear, and only a few cellular factors have been shown to bind PA and affect polymerase function (44–46), despite bioinformatics indicating many possible candidates (47–49).

The H5N1 clade 2.2.1 viruses that are unique to Egypt generally carry the human adaptation PB2-E627K mutation in the field and have no history of reassortment during long-term dissemination. This gave us the opportunity to identify novel human adaptation mutations in the polymerase complex, other than PB2-E627K, that have accumulated during viral evolution in nature, especially in PA and PB1.

In this study, we traced the evolution of the PA gene in clade 2.2.1 influenza viruses and found that multiple PA mutations accumulated during viral evolution in the field. Several of these PA mutations (i.e., A448E, S388R, and E18G) acted cooperatively to increase polymerase activity and viral replication in both avian and human cells (Fig. 5 to 7) and to enable an increase in viral growth and virulence in mice (Fig. 8). Almost all the contemporary clade 2.2.1 viruses in this study have retained A448E and S388R (Fig. 3), indicating their possible role in establishing contemporary clade 2.2.1.2 virus infections in poultry, leading to a more fit phenotype to infect humans. Indeed, the A448E/S388R signature mutation had more prominent effects on improving polymerase and replication activity in human cells than in avian cells, especially in human cells at the lower temperature of 33°C (Fig. 5 [Fig F6]to 7). In addition, the A448E, S388R, and E18G mutations had a replication-enhancing effect in viruses in the clade 2.2.1 background carrying PB2-627K, implying cooperative polymerase activity with PB2-E627K. These results suggested that the continuing dissemination of clade 2.2.1 viruses in the field has allowed these viruses to accumulate multiple PA mutations that enabled higher polymerase activity by themselves and in concert with PB2-E627K to fine-tune the viral polymerase function for host range expansion. Although not all phylogeny-associated PA mutations identified in this study were involved with polymerase activity, this may be because mutations arise stochastically and may not necessarily be associated with an adaptive change.

The PA protein is structurally separated into N-terminal (PA-N) and C-terminal (PA-C) domains, which are connected by a narrow linker (residues 257 to 276) (33). PA-N (residues 1 to 256) is the major functional part of the PA protein and contains the cap-snatching endonuclease, the protease active sites (50–53), and a bipartite nuclear localization signal (residues 124 to 139 and 186 to 247) (54). PA-C (residues 277 to 716) interacts with PB1 to form the core structure of the polymerase complex (55, 56). In addition, PA-C contains an extended loop, the PA arch (residues 367 to 396), which forms a 5′ vRNA binding site (57), and an interaction site with cellular polymerase II (Pol II) (58) to take 5′-capped primers from nascent Pol II transcripts to transcribe viral mRNA.

The PA-S388R and -A448E mutations, which were identified in this study as main contributors to the higher polymerase activity of contemporary clade 2.2.1.2 viruses, were both localized in PA-C. In the crystal structure, PA residue 388 lies within the PA arch domain, which forms an integral part of the 5′ vRNA binding site together with the adjacent PB1 β-hairpin domain (57). In our model, PA-S388, but not -R388, participated in a hydrogen bond network with PB1. This indicated that the S388R substitution created flexibility at this position, implying the possible association of the basic amino acid with the vRNA promoter. A comprehensive evolutionary analysis reported S388N to be one of the evolution-associated mutations in H7N9 virus (59), which was presumably associated with its host range and infection of humans, although its biological significance has not been tested. Thus, S388R may affect viral polymerase activity by altering the interaction between the polymerase complex and the viral RNA promoter.

PA residue 448 is in helix α16 in the polymerase complex structure and is one of the key residues that make direct contact with the Pol II C-terminal domain for transcription (58). Helix α16 is part of a major platform that interacts with the PB1 thumb domain (residues 398 to 498) (60) and includes several residues that are essential during replication (i.e., R442 and R443) (56). In our apo-form model, A448E created a new hydrogen bond to N444, probably stabilizing the helix α16 structure. A previous report showed that mutation of PA residue 448 can change the helix α16 structure by breaking its interaction with an adjacent helix, thereby interfering with RNA synthesis by the trimeric polymerase (55). Therefore, the A448E substitution may affect polymerase activity by changing the core structure of the polymerase complex or its interaction with cellular Pol II.

The effect of PA-E18G was different from those of the above-described two mutations. PA-E18G is in PA-N and was characterized here as an auxiliary mutation for increasing polymerase activity and unique to an EG13 virus strain. PA-E18G was found in H1N1 pdm09 virus during a marmoset infection (nonhuman primate model) (61) and during serial passages in mice (62). Therefore, the E18G substitution may produce more fit polymerase activity in specific host species, but this mutation may not be associated with H5N1 evolutionary dynamics since it has not been found in any other clade 2.2.1 virus. Residue 18 is within the first 182 PA amino acids that are in PA-X. Therefore, E18G may also affect the function of PA-X. Alternatively, the three PA mutations identified in this study may alter polymerase activity by interacting with cellular host factors since they were all exposed on the polymerase surface. Collectively, these results reinforced the hypothesis that adaptive PA mutations may have multiple functions in expanding the viral host range (35). At present, the exact mechanism(s) by which PA mutations enhance polymerase activity remains unknown, and further investigation is needed.

The number of human H5N1 cases has declined considerably in Egypt since 2017. Nonetheless, the persisting circulation of clade 2.2.1 viruses in poultry poses an ongoing risk to humans (26). Contemporary clade 2.2.1.2 viruses have now cocirculated with H9N2 and H5N8 viruses in the field, which has provided a potential opportunity for reassortment to generate novel viruses in nature (6). The PB2 genes from clade 2.2.1.2 and H9N2 G1-A/B subclade viruses in Egypt have commonly acquired PB2-E627K and PB2-E627V, respectively (13, 18), both of which have been reported to confer improved replication and an expanded host range. Therefore, the signature PA-S388R/A448E mutation, in particular paired with PB2-627K/V, may be a viral marker for severe risk to human health in the Middle East, centered in Egypt. Our results highlighted the need for continual tracing of the evolutionary dynamics of AI viruses in Egypt and close monitoring of possible genetic mutations and reassortment in these viruses to reduce the public health risk.

## MATERIALS AND METHODS

### Ethics statement.

Nine-day-old embryonated chicken eggs were purchased from Shimizu Laboratory Supplies, Japan. All animal experiments were conducted in compliance with Japanese legislation (Act on Welfare and Management of Animals, 1973, revised in 2012) and guidelines under the jurisdiction of the Ministry of Education, Culture, Sports, Science, and Technology in Japan. Animal care, housing, feeding, sampling, observation, and environmental enrichment were approved by the Animal Experiment Committee of the Kyoto Prefectural University of Medicine (approval numbers M29-554 and M30-60).

### Biosecurity and biosafety.

All experiments with live H5N1 viruses were performed at enhanced biosecurity level 3^+^ (BSL3^+^) at the Kyoto Prefectural University of Medicine. All studies with recombinant DNA were conducted under the relevant laws in Japan and approved by the Biological Safety Committee of the Kyoto Prefectural University of Medicine (approval number 30-104) after risk assessments were conducted by the Living Modified Organisms Committee of the Kyoto Prefectural University of Medicine and, when required, by the Ministry of Education, Culture, Sports, Science, and Technology of Japan.

### Cells and viruses.

293T cells were obtained from the Riken BioResource Center Cell Bank. Human bronchial epithelial (Calu-3) cells and chicken fibroblast (DF-1) cells were obtained from the American Type Culture Collection. The cells were maintained in Dulbecco’s modified Eagle’s medium (DMEM) with 10% fetal calf serum (FCS). AI viruses A/duck/Egypt/D1Br/2007 (denoted D1 here) and A/chicken Egypt/CL69/2013 (denoted EG13 here) are representative ancestral and contemporary strains of H5N1 clade 2.2.1, respectively. The details of the two viruses were described previously (6, 24, 63). All recombinant H5N1 viruses were propagated once in 9-day-old embryonated eggs and purified by ultracentrifugation as described previously (24).

### Generation of recombinant viruses by reverse genetics.

Recombinant viruses were generated using a plasmid-based reverse-genetics system in the EG13 and D1 genetic backgrounds as described previously (6, 24, 63). The mutations identified in this study were introduced into plasmids using PCR-based site-directed mutagenesis. All propagated viruses were completely sequenced to ensure the absence of unwanted mutations. Virus titration was performed by measuring focus-forming units (FFU) in focus-forming assays on MDCK cells as described previously (19, 24).

### Minigenome assays.

293T and DF-1 cells were transfected with pCXN2 plasmids carrying the PB2, PB1, PA, and NP genes of EG13 or D1 and a human or chicken polymerase I-driven plasmid expressing firefly luciferase as described previously (18, 64). A plasmid expressing *Renilla* luciferase was also cotransfected to measure transfection efficiencies. The transfected cells were incubated at 33°C or 37°C and collected with cell lysis buffer at 24 h posttransfection. The firefly luciferase activities were expressed relative to the *Renilla* luciferase activity.

### Phylogenetic analysis.

PA gene sequences of the H5N1 clade 2.2.1 virus strains, isolated in Egypt in 2006 to 2015, were obtained from the GISAID database (http://www.gisaid.org). A phylogenetic tree was reconstructed using MEGA6 software for the neighbor-joining method with the EG13 and D1 nucleotide sequences. The confidence level for the phylogenetic tree was calculated by performing 1,000 bootstrap replicates.

### Immunoprecipitation assays of the polymerase complexes.

293T cells were transfected with pCXN2 plasmids carrying the PB2-Flag, PB1, and PA genes of EG13 or D1. At 48 h posttransfection, cells were harvested in Tris lysis buffer, and immunoprecipitation assays were carried out as described previously (13). Purified polymerase proteins were identified by Western blotting as described below.

### Western blotting.

293T cells were transfected with pCXN2 plasmids carrying the PA genes of EG13 and D1 or these PA genes carrying the mutations in this study, and the cells were harvested with sample buffer at 16 h posttransfection. The samples were boiled, analyzed by SDS-PAGE, and transferred onto a polyvinylidene difluoride membrane (Millipore). The PA proteins were detected with anti-influenza virus PA antibody (GeneTex) and horseradish peroxidase (HRP)-conjugated secondary antibody. The Amersham ECL Select Western blotting reagent was used for band visualization. The band intensities were quantified using Amersham Imager 600 analysis software (GE Healthcare).

### Viral infection of cell cultures.

Calu-3 cells and DF-1 cells were seeded on a 24-well plate (90% confluent) and infected with the indicated viruses at multiplicities of infection (MOIs) of 0.03 and 0.003, respectively. After 1 h of incubation at 37°C, the cells were washed twice with phosphate-buffered saline (PBS), maintained in DMEM–F-12 medium containing 0.2% bovine serum albumin (BSA), and incubated at 37°C or 33°C. The supernatants were collected at the indicated times, and progeny virus titers were measured by focus-forming assays as described above.

### Viral infection of mice.

Four- to five-week-old female BALB/c mice (Japan SLC), under mixed anesthesia (medetomidine-butorphanol-midazolam), were inoculated intranasally with 50-μl samples of 10-fold serial dilutions (10^1^ to 10^5^ FFU) of viruses in PBS. The body weight and survival of each mouse were monitored daily for 2 weeks. Mice that lost more than 30% of their original weight within a few days were humanely euthanized. Lungs of mice infected with 10^3^ FFU of virus were collected at 6 dpi, and virus FFU titers were assayed. Immunohistochemical staining of the viral H5 antigen was performed as described previously (18, 24).

### Homology modeling of the polymerase complex.

The transcription preinitiation form and the apo form of the D1/PA trimeric polymerase complex with the indicated PA forward mutations were modeled using the published crystal structure of the influenza A virus polymerase complex (PDB accession numbers 6RR7 and 6QPF, respectively). MOE was used for software, and most of the modeling conditions were described previously (18, 19, 64). In a change from previous studies, the number of side chain samples was set at 100.

### Statistical analysis.

Statistical analysis was carried out using GraphPad Prism version 6 software (GraphPad Software). Statistically significant differences between virus pairs were determined by analysis of variance (ANOVA) with Tukey’s multiple-comparison test.
